# Supplementary material for: Dissecting the Binding Affinity of Anti-SARS-CoV-2 Compounds to Human Transmembrane Protease, Serine 2: A Computational Study
Source: Int J Mol Sci. 2025 Jan 11;26(2):587. doi: 10.3390/ijms26020587 (PMC11766390; doi:10.3390/ijms26020587)
Supplement: Supplementary file 1 [file ijms-26-00587-s001.zip › Supplementary materials.pdf]

Table S1. Residue binding free energy (RBFE) values and energy components (kJ/mol) for TMPRSS2 bound to 12 compounds (see the file Table_S1.xlsx).

Table S2. RBFE values and energy components (kJ/mol) for TMPRSS2 residues significantly impacting binding affinity (RBFE exceeding ±3.0 kJ/mol) for at least one neutral compound or forming specific electrostatic-related interactions (see the file Table_S2.xlsx). The positive and negative hotspots are highlighted in green and red, respectively.

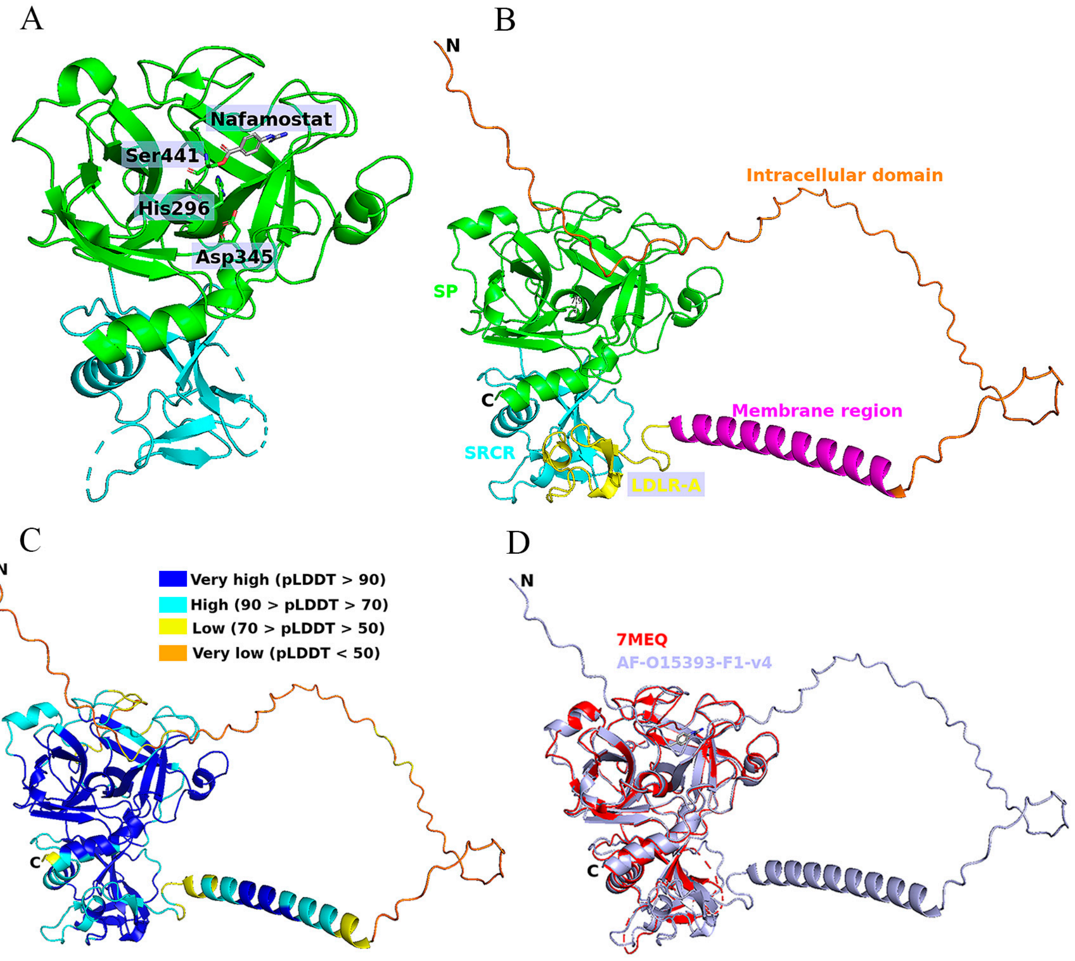

Figure S1. Structural comparison of the experimentally determined extracellular domain of TMPRSS2 and the AlphaFold2-predicted full-length model. (A) Crystal structure of the TMPRSS2 extracellular domain in complex with nafamostat (PDB ID: 7MEQ). The catalytic triad (Ser441–His296–Asp345) and nafamostat are shown in the stick model, with O and N atoms in red and blue, respectively, and C atoms in green (protein) and gray (ligand). (B) Domain-colored full-length TMPRSS2 structure predicted by AlphaFold2 (AlphaFold database ID: AF-O15393-F1-v4). The extracellular domain comprises LDLR-A (low-density lipoprotein receptor type-A), SRCR (scavenger receptor cysteine-rich), and SP (serine protease) subdomains. (C) Confidence-colored full-length AlphaFold2 structure. Predicted local distance difference test (pLDDT) scores indicate very low confidence (pLDDT < 50) for the intracellular domain, and high (70 < pLDDT < 90) to very high confidence (pLDDT >

90) for other domains. (D) Superimposed X-ray structure (PDB ID: 7MEQ) and AlphaFold2-predicted structure (AF-O15393-F1-v4), showing excellent alignment of the extracellular domain (Cα RMSD of 0.5 Å for this domain).

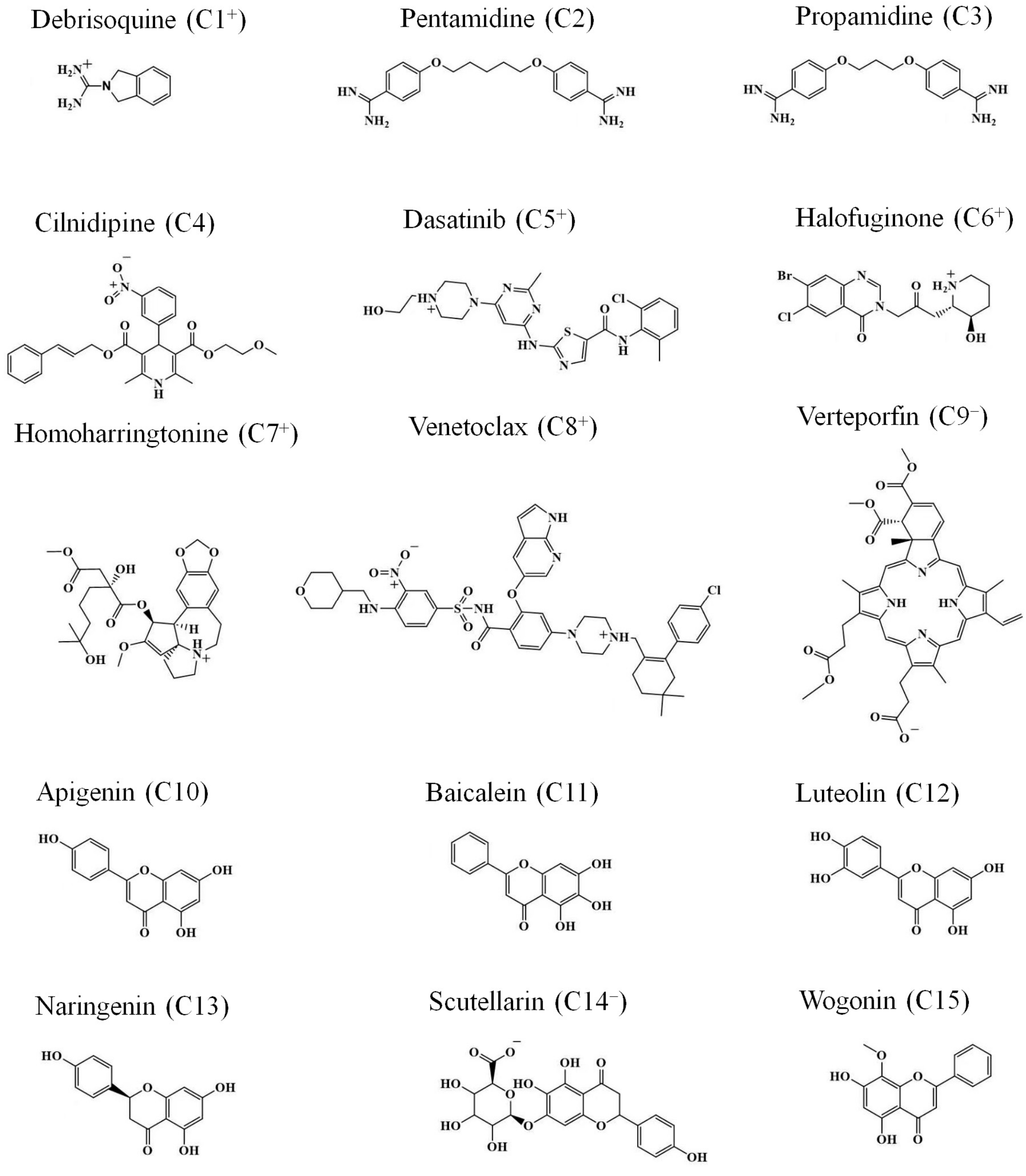

Figure S2. 2D structures of the 15 anti-SARS-CoV-2 compounds acting via TMPRSS2-dependent mechanisms. Compound names and identifiers (in parentheses) are labeled. Corresponding ZINC IDs are listed in Table 1 of the main text.

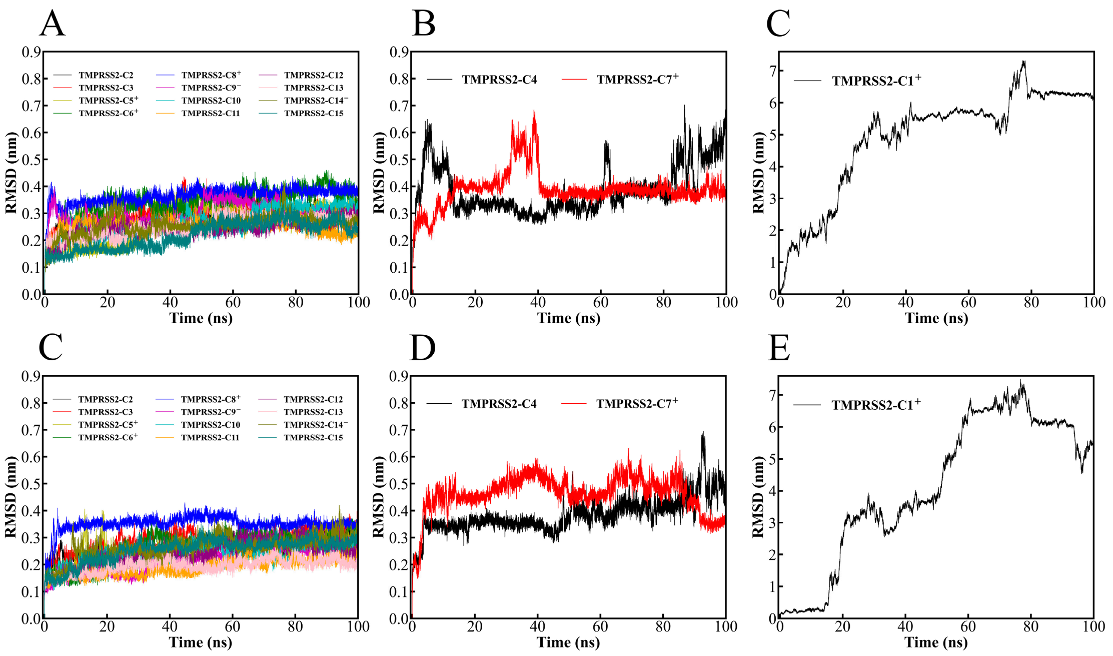

Figure S3. Complex RMSD values calculated using TMPRSS2 Cα atoms and compound heavy atoms from two additional 100 ns MD simulation replicates. (A–C) The RMSD curves for the second replicates, showing complexes with values below 0.5 nm, briefly exceeding 0.5 nm, and consistently exceeding 0.5 nm, respectively. (D–F) RMSD curves for the third replicates, showing the same classification of complexes as in (A–C).

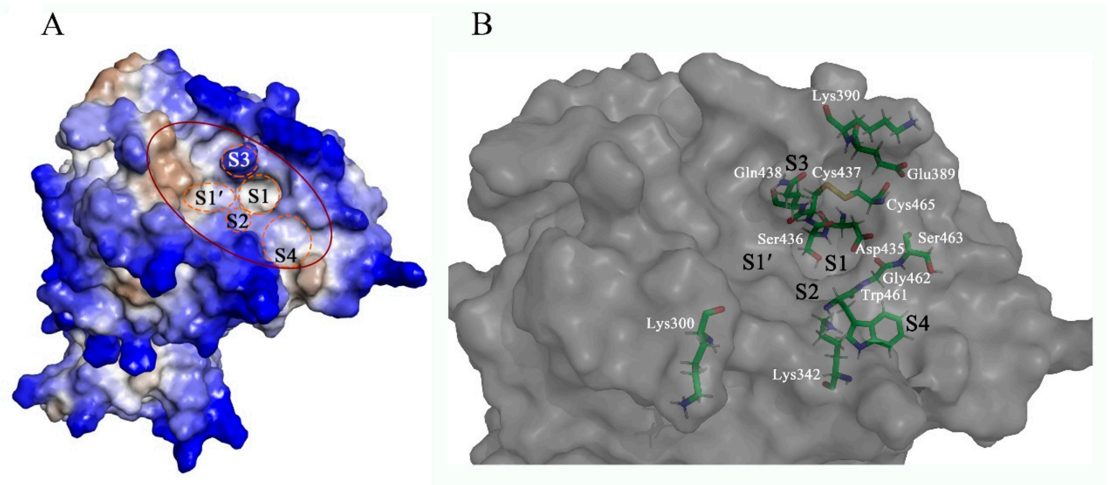

Figure S4. Surface representation of TMPRSS2. (A) shows the substrate-binding cavity (SBC), comprising the pockets/subsites S4, S3, S2, S1, and S1'. The protein surface is colored by hydrophobicity, from blue (most hydrophilic) to brown (most hydrophobic surface). (B) highlights the residues within/near the SBC that significantly impact the binding affinity or participate in specific electrostatic-related interactions with certain compounds. The protein surface is colored gray, with residues shown in the stick model. Atom colors: C (green), O (red), N (blue), S (yellow), and H (white).

File S1. Python script for constructing free energy landscapes (FELs) of the 12 TMPRSS2-compound complexes (see the file File_S1.py).
